# Fatal subarachnoid hemorrhage following ischemia in vertebrobasilar dolichoectasia

**DOI:** 10.1097/MD.0000000000004020

**Published:** 2016-07-08

**Authors:** Arseny A. Sokolov, Shakir Husain, Roman Sztajzel, Alexandre Croquelois, Johannes A. Lobrinus, David Thaler, Claudio Städler, Hansjörg Hungerbühler, Valeria Caso, Gabriel J. Rinkel, Patrik Michel

**Affiliations:** a Stroke Center, Service de Neurologie, Département des Neurosciences Cliniques, Centre Hospitalier Universitaire Vaudois (CHUV) and University of Lausanne, Lausanne, Switzerland; b Department of Interventional Neurology and Stroke, Institute of Neurosciences, Saket City Hospital, New Delhi, India; c Service de Neurologie, Département des Neurosciences Cliniques, Hôpitaux Universitaires de Genève (HUG), Geneva, Switzerland; d Service de Neurologie, Fondation Hopale, Berck-sur-Mer, France; e Service de Pathologie Clinique, Hôpitaux Universitaires de Genève (HUG), Geneva, Switzerland; f The Comprehensive Stroke Center, Tufts University Medical Center, Boston, MA; g Servizio di Neurologia, Ospedale Regionale di Lugano, Lugano; h Neurologische Klinik, Kantonsspital Aarau, Aarau, Switzerland; i Stroke Unit and Division of Internal and Cardiovascular Medicine, Ospedale Santa Maria della Misericordia, Perugia, Italy; j Department of Neurology and Neurosurgery, Brain Center Rudolf Magnus, University Medical Center Utrecht, Utrecht, The Netherlands.

**Keywords:** dolichoectasia, ischemia, stroke, subarachnoid hemorrhage, vertebrobasilar

## Abstract

Vertebrobasilar dolichoectasia (VBD) is a chronic disorder with various cerebrovascular and compressive manifestations, involving subarachnoid hemorrhage (SAH). Occurrence of SAH shortly after worsening of clinical VBD symptoms has occasionally been reported. The goal of the study was to examine this association, in particular its pathophysiology, clinical precursor signs, time course, and outcome.

To this end, in a retrospective multicenter study, we analyzed 20 patients with VBD and SAH in regard to preceding clinical symptoms, presence of vertebrobasilar thrombosis and ischemia, outcome and neuropathological correlates.

Median age of the 7 female and 13 male patients was 70 years (interquartile range [IQR] 18.3 years). Fourteen patients (70%) presented with new or acutely worsening posterior fossa signs at a median of 3 days prior to SAH (IQR 2, range 0.5–14). A thrombus within the VBD was detected in 12 patients (60%). Thrombus formation was associated with clinical deterioration (χ^2^ = 4.38, *P* = 0.04) and ponto-cerebellar ischemia (χ^2^ = 8.09, *P* = 0.005). During follow-up after SAH, 13 patients (65%) died, after a median survival time of 24 hours (IQR 66.2, range 2–264 hours), with a significant association between proven ponto-cerebellar ischemia and case fatality (χ^2^ = 6.24, *P* = 0.01).

The data establish an association between clinical deterioration in patients with VBD, vertebrobasilar ischemia, and subsequent SAH. Antithrombotic treatment after deterioration appears controversial and SAH outcome is frequently fatal. Our data also indicate a short window of 3 days that may allow for evaluating interventional treatment, preferably within randomized trials.

## 1 Introduction

Dolichoectasia refers to winding and dilated arteries,^[1,2]^ and is associated with arterial hypertension and genetic predisposition.^[3,4]^ Estimations of dolichoectasia prevalence range from 0.1 to >12 %, with a trend to higher prevalence in more recent studies,^[3,5,6]^ likely due to increased life expectancy but also more widespread use of imaging. Dolichoectasia is predominantly diagnosed around the age of 60,^[7]^ and more often affects the posterior than anterior cerebral circulation.^[1,8]^ Vertebrobasilar dolichoectasia (VBD) may cause brainstem and cranial nerve compression along with corresponding symptoms such as bilateral or isolated oculomotor deficits,^[9]^ pyramidal and cerebellar signs,^[10,11]^ trigeminal neuralgia,^[7]^ cranial nerve deficits,^[12]^ and hydrocephalus.^[5,13]^

Even more important, ischemia represents a central concern in VBD and occurs usually in brainstem, pons,^[12,14,15]^ or the thalamus.^[14]^ Dolichoectasia is present in 12% to 17 % of patients with cerebral ischemia as indicated by MR angiography, and VBD is related to 7% of all posterior circulation ischemic strokes.^[2,6,8,16]^ Another major complication in patients with VBD are subarachnoid and intraparenchymal hemorrhage,^[5,12,13,15,17–21]^ with an annual prospective risk between 1% and 2.5%.^[22]^ Previous reports suggest subarachnoid hemorrhage (SAH) in VBD may rapidly follow ischemia.^[15,17–25]^ However, the link between clinical change, vertebrobasilar thrombosis, posterior circulation ischemia, and SAH remains largely speculative.

The goal of this study was to assess temporal and statistical associations between SAH and acute clinical worsening, thrombosis, and ischemia in patients VBD, the role of antithrombotic treatment, as well as pathophysiology and outcome of SAH.

## 2 Methods

### 2.1 Participants

Between 1997 and 2012, all consecutive patients hospitalized at the principal investigator center (Centre Hospitalier Universitaire Vaudois, CHUV, Lausanne, Switzerland) who presented with SAH and no other potential cause than VBD were included in this retrospective study. Patients were identified on daily ward rounds in neurology, neurosurgery, and intensive care (n = 7) and from the hospital's database (n = 2). Written informed consent was obtained. Two patients were described in a previous report.^[20]^ In addition, nonconsecutive patients with SAH due to VBD were identified in other cerebrovascular centers in Switzerland, Italy, India, the United States, and the Netherlands. The study was approved by the Institutional Ethics Review Board at the CHUV in Lausanne, Switzerland.

### 2.2 Clinical assessment

We analyzed past medical history, cerebrovascular risk factors and the time interval between VBD diagnosis and the index admission, if the diagnosis was known prior to hospitalization. Neurological symptoms at initial diagnosis of VBD and at admission were evaluated. In patients with acute clinical deterioration preceding SAH, the time interval between symptom onset and occurrence of SAH was recorded. Survival rate and possible associations between different clinical presentations and outcome were evaluated. Long-term and acute antithrombotic treatment strategies were also assessed.

### 2.3 Radiological and pathological data

**Figure 1:**
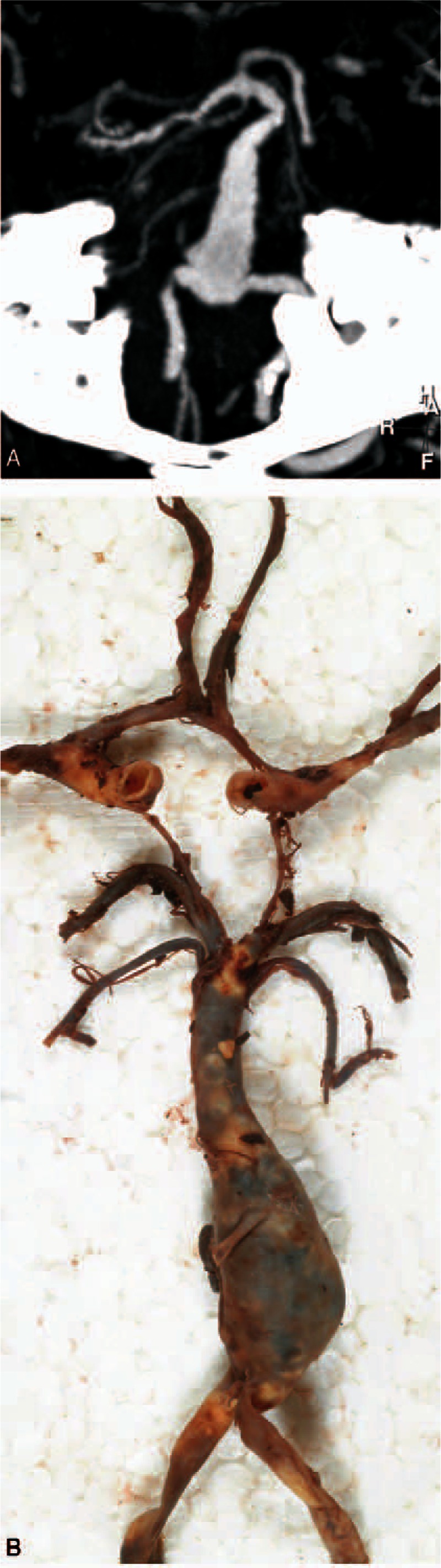
Clinico-pathological findings. (A) Axial CT angiogram and (B) pathological specimen show vertebrobasilar dolichoectasia (VBD), with its typical elongation and tortuosity. The basilar trunk is predominantly affected by VBD. However, as in these cases, dilated caliber of the vertebral arteries can also occur. CT = computed tomography, VBD = vertebrobasilar dolichoectasia.

**Figure 2:**
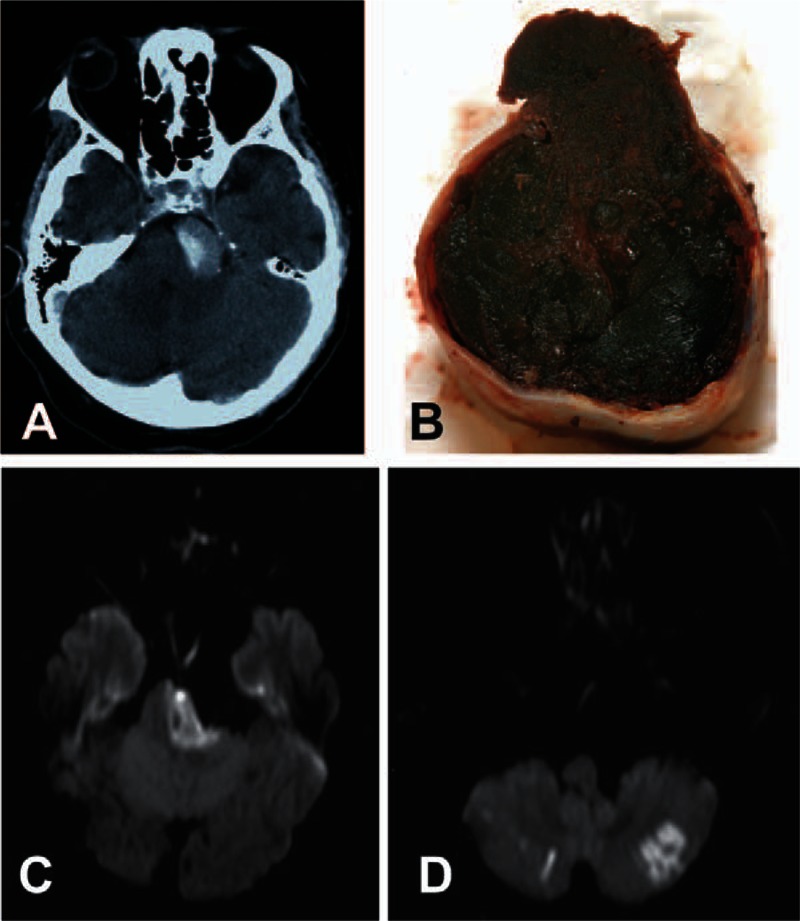
Thrombosis and stroke in dolichoectasia. (A) Axial CT angiogram revealing thrombosis within the dilated basilar trunk of a vertebrobasilar dolichoectasia (VBD). (B) Pathological examination showed multilayer acute-on-chronic thrombi. Only a small residual lumen could be detected. Rupture site (upper segment of the image) was located at the same level as the thrombus. (C) Axial diffusion-weighted MRI showing infarction of the left pons with pontine compression by the dolichoectatic basilar trunk. (D) Diffusion-weighted MRI with infarction in both cerebellar hemispheres. CT = computed tomography, MRI = magnetic resonance imaging, VBD = vertebrobasilar dolichoectasia.

VBD and intraluminal thrombus formation was diagnosed by computed tomography angiography (CTA, Fig. 1A). Initial SAH diagnosis was established by CT in 19 patients and through autopsy in one. During the first days of hospitalization, most of the patients also underwent magnetic resonance imaging (MRI) to examine signs of acute or subacute ischemia (Fig. 2). Collected radiological variables also included leukoaraiosis and other vascular abnormalities. Six of the 9 CHUV patients underwent autopsy.

### 2.4 Statistical analysis

Median values and interquartile ranges were calculated for age, symptom duration, and time on prophylactic antithrombotic treatment, as well as survival after SAH. Proportions reflected in percentage values were calculated for the presence or the absence of specific findings, treatments, and outcomes in the study population. Chi-square statistics with Yates correction were used to calculate associations between thrombosis and ischemia, ischemia and preventive antithrombotic therapy, and between fatal outcome and clinical presentation (with/without chronic VBD symptoms; with/without acute clinical change prior to SAH), thrombosis and ischemia. All tests of significance were 2-tailed.

## 3 Results

### 3.1 Clinical presentation

Among the 7 female and 13 male patients, median age was 70 years (interquartile range [IQR] 18.3 years, range 47–93 years). A total of 65% of the patients had known VBD, diagnosed at a median of 17 months prior to SAH (IQR 45 months, range 0.4–144 months). At initial VBD diagnosis, bulbar symptoms (especially dysarthria and dysphagia) prevailed, as well as other posterior fossa symptoms, such as ataxia and vertigo. Diverse cranial nerve deficits were present, most often unilateral facial and abducens nerve palsies; hemiplegia and nystagmus were less common (Table 1A).

**Table 1:**
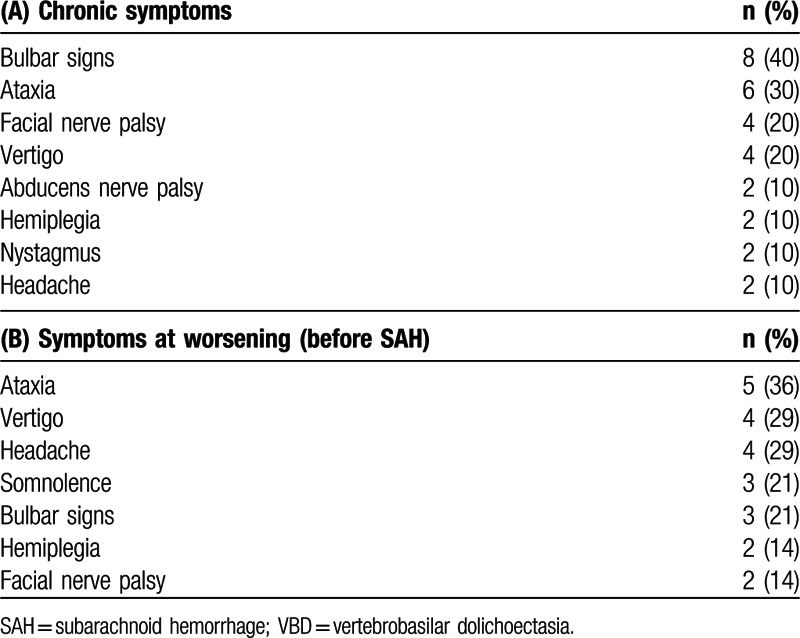
Clinical presentation of vertebrobasilar dolichoectasia: (A) clinical picture along with number and percentage of patients affected at initial diagnosis and during the course of chronic evolution of vertebrobasilar dolichoectasia (VBD). (B) Newly occurred or deteriorated symptoms at index admission, with number of patients and percentage referring to all patients with clinical change prior to SAH (n = 14). Only symptoms observed in at least 2 patients reported.

New clinical signs or acute deterioration of existing deficits prior to SAH were observed in 70% of patients (n = 14), preceding SAH by a median of 3 days (IQR 2 days, range 0.5–14 days). Predominant symptoms at worsening were ataxia, vertigo, headache, alteration of consciousness, and bulbar signs (Table 1B). One patient each presented with downbeat nystagmus, a locked-in syndrome or Horner's syndrome.

Six patients had sudden-onset SAH without preceding clinical signs. In 4 of these patients, VBD had been previously diagnosed. In only 1 patient was SAH the initial presentation of VBD. Another patient was admitted with sudden second SAH due to known VBD, having survived the initial one. Examining the etiology of all SAH patients admitted over the study duration (16 years, n = 873) at the CHUV, we found that 1% (n = 9) were due to VBD.

### 3.2 Vertebrobasilar thrombosis and ischemia

A thrombus within the VBD was detected (radiologically and/or pathologically; Fig. 2) in 60% of all patients, and in 79% of patients with acute clinical change shortly before SAH. In turn, 92% of patients with a VBD thrombus presented new or worsened neurological signs. Hence, the presence of thrombosis was associated with clinical change before SAH (χ^2^ = 4.38, *P* = 0.04). Brainstem, pontine, or cerebellar infarction was confirmed in 45% of all patients on MRI (Fig. 2). Ischemia was present in 64% of patients with change in neurological status prior to SAH, and in 75% of patients with VBD thrombosis. There was a significant association between basilar thrombosis and radiologically detectable infarction (χ^2^ = 8.09, *P* = 0.005).

### 3.3 Cardiovascular risk factors

All but 1 patient (95%) were known to have arterial hypertension. A total of 45% of the patients were smokers, 20% diabetic, and 50% had dyslipidemia. Leukoaraiosis was present in 84% (data not available in 1 patient). Supplementary radiological findings and/or previous medical history revealed concomitant intra- or extracranial vascular and organ abnormalities in 65% of the patients, such as anterior cerebral circulation aneurysms or dolichoectasia, aortic aneurysms and dilatation, and polycystic kidney disease. None of the patients had a specific diagnosis of an underlying collagen or other genetic disorder, but none underwent genetic testing.

### 3.4 Treatment and outcome

Early case fatality in this series was 65%. Most patients died within 1 day after SAH (median 24 hours, IQR 66.25, range 2–264 hours). Three patients survived until 9 to 11 days post-SAH. One patient had already had an initial SAH due to VBD 2 months prior to the index SAH. Neither hemorrhage was preceded by clinical symptoms, with the second event being fatal. Preceding posterior circulation ischemia was significantly associated with fatal outcome (χ^2^ = 6.24, *P* = 0.01), whereas vertebrobasilar thrombosis alone was not (χ^2^ = 2.65; *P* > 0.05). Survival rate in patients with chronic VBD symptoms before SAH was not significantly different to that in patients without known VBD (31% vs 43%, respectively; χ^2^ = 0.002, *P* > 0.05). In our patients, except for headache, every symptom acutely appearing or worsening before SAH (Table 1B) was associated with ponto-cerebellar ischemia and fatal outcome. Overall, recent clinical deterioration before SAH did not influence survival rate (35% vs 33%, respectively; χ^2^ = 0.01, *P* > 0.05). The following analysis on therapeutic management is therefore presented across all patients.

Prior to the index event, 45% of the patients were treated with antiplatelet agents (7 patients on aspirin, 1 on clopidogrel, and 1 on both) for a median duration of 60 months (IQR 24, range 6–180 months). Three patients were anticoagulated with vitamin K antagonists for a median duration of 3 years (IQR 6.84, range 0.33–14 years), with 1 taking aspirin concurrently. Ischemia in our VBD patients was present irrespective of preventive antithrombotic therapy (χ^2^ = 0.25, *P* > 0.05). In regard to acute management strategies, following clinical deterioration, aspirin was introduced in 6 patients at a median of 5 days (IQR 4, range 2–7 days), and therapeutic heparin in 3 patients (all had been on long-term aspirin) at a median of 2 days (IQR 1.75, range 0.5–4 days) before SAH. Given the short interval between admission and SAH, patients on chronic aspirin that were anticoagulated after clinical deterioration were considered being on double antithrombotic treatment at time of SAH.

All 5 patients on double antithrombotic treatment died. Of the 10 patients on antiplatelet drugs alone, 50% survived the SAH. One patient was on a vitamin K antagonist alone at time of SAH and survived. Four patients did not receive any antithrombotic treatment and 3 of them passed away (only 1 of them with prior clinical deterioration, the other 3 with sudden-onset SAH). The patient who survived underwent complex endovascular treatment.

### 3.5 Pathological examination

Autopsy in 6 patients from the CHUV confirmed SAH resulting from ruptured VBD (Fig. 1B). All autopsied patients had acute brainstem ischemia and thrombosis within the VBD. In 1 patient, thrombosis was diagnosed through pathological examination only. We found multilayer thrombi indicating turbulent blood flow with stasis close to the arterial wall as the principal mechanism for thrombus formation. Most of the patients had signs of acute-on-chronic thrombosis. Despite substantial enlargement of the artery, only a small residual lumen was detectable in these patients (Fig. 2B).

In terms of VBD pathogenesis, degeneration of the internal elastic lamina, intramural hematoma, defects of the media smooth muscle layer and arterial wall fibrosis were observed. There was no significant burden of atherosclerotic changes. Although, in general, rupture site was at the same level as the thrombus (Fig. 2B), due to extensive chronic wall abnormalities and limited transformation of collagenous tissue following ischemia, pathological examination did not allow clarifying whether vessel rupture was primarily due to ischemia of the arterial wall through thrombus compression or occlusion of vasa vasorum.

## 4 Discussion

Our multicenter retrospective study indicates that SAH in VBD is often heralded by new or worsened symptoms from ponto-cerebellar ischemia associated with endoluminal thrombus formation, confirming previous accounts of potentially fatal SAH following clinical and radiological signs of posterior circulation ischemia in patients with VBD.^[15,17–21,23–25]^ VBD appears to represent a rare cause of SAH (∼1% of patients admitted with SAH in the CHUV cohort); however, it is quite a precarious one, given a case fatality of 65% which corresponds to nearly twice the SAH fatality in general.^[26]^

Ischemia in VBD is thought to be caused by a combination of reduced blood flow, thrombus formation, thromboembolism, perforator occlusion, and shear stress.^[3,6,15,27]^ Most of our pathologically examined patients had multilayer acute-on-chronic thrombi indicating progressive thrombosis due to perturbed blood flow. The mechanisms leading to rupture of VBD and SAH may involve decreasing arterial wall resistance from progressive dilatation (Laplace's law), absence of protective intimal thickening within the VBD, inflammation and ischemia of the vessel wall through local effects by the thrombus or occlusion of vasa vasorum.^[28]^ Artery wall thinning due to spontaneous or induced lysis may have also contributed to rupture and SAH, as previously suggested.^[19]^ Vertebrobasilar thrombosis and ponto-cerebellar ischemia were tightly linked to acute clinical change. In those 3 patients with acute clinical symptoms but without vertebrobasilar thrombosis, we assume that symptoms preceding SAH resulted from compression of adjacent structures by VBD enlargement. This agrees well with previous data on increased risk of SAH in patients with progressive VBD enlargement.^[22]^

The present study reveals that ponto-cerebellar ischemia closely preceding SAH in VBD is a strong predictor of case fatality. This poor outcome in the vast majority of our patients indicates that combination of acute ischemia and hemorrhage in VBD is particularly perilous. Strategic localization of this disease in the brainstem with its vital structures may also contribute to the high case fatality rate. Management of patients with VBD remains controversial due to the lack of randomized controlled trials. For ischemia in VBD, primary prevention by aspirin and acute treatment or secondary prevention by anticoagulation represent the only conventional treatment options.^[2]^ Some authors found an increased risk of hemorrhagic complications^[13]^ or ineffectiveness in preventing ischemia in VBD,^[29]^ whereas others suggest a possible survival benefit.^[30]^ A recent review is unfavorable of secondary prevention by anticoagulation due to the high risk of hemorrhage.^[2]^ As to acute phase management, while limited by inclusion criterion and small sample size, the present observations indicate that initiation of anticoagulation or double antithrombotic treatment may not be useful. Long standing antiplatelet therapy may have some beneficial effect. Among other effects, this may agree with recent radiological and histopathological data suggesting attenuation of aneurysm wall inflammation through low-dose aspirin.^[31]^ Additional prospective research is required to clarify strategies for conventional and interventional treatments.

The observed interval of about 3 days between symptom onset and SAH in patients with clinical change may provide an opportunity for careful evaluation and planning of endovascular treatment or surgery. Endovascular reconstruction and embolization is achieved through combining stents, coils, and flow diverters.^[32,33]^ Surgical interventions include parent vessel occlusion,^[34]^ decompression and repositioning,^[11]^ and vein bypasses.^[35]^ Interventions in VBD remain experimental and associated with considerable rates of severe complications: 10% to 30 % for endovascular and 12% to 15 % for surgical procedures.^[32–34]^ A long-term follow-up study on stent-assisted endovascular repair showed satisfactory results in 8 out of 9 patients with VBD, including 2 suffering from acute SAH, with 1 of the 9 patients passing away 2 weeks after intervention.^[36]^ In our study, the only patient undergoing complex endovascular treatment following spontaneous SAH survived, with excellent evolution.

Limitations of our study include the partially nonconsecutive collection of patients within multiple centers, moderate sample size and heterogeneous treatment approaches, somewhat limiting statistical inferences.

## 5 Conclusion

In summary, this clinico-pathological study confirms a pathophysiological association between SAH and preceding acute clinical deterioration due to vertebrobasilar thrombosis in patients with VBD. As compared to other SAH etiologies, case fatality due to SAH in VBD is twice as high, and higher in those with concurrent posterior circulation ischemia. Despite limitations, the data suggest initiation of anticoagulation after clinical worsening in VBD may not be useful. In addition, the findings reveal a short therapeutic window of 3 days during which endovascular or surgical intervention might be evaluated on an individual basis, until randomized interventional data become available.

## Acknowledgments

The authors wish to thank Mireille Nya Buvelot for her assistance with retrieval of patient records.
